# The Association of Disease Activity, BMI and Phase Angle with Vitamin D Deficiency in Patients with IBD

**DOI:** 10.3390/nu11112583

**Published:** 2019-10-26

**Authors:** Maria Chiara Mentella, Franco Scaldaferri, Marco Pizzoferrato, Antonio Gasbarrini, Giacinto Abele Donato Miggiano

**Affiliations:** 1 UOC di Nutrizione Clinica, Area Medicina Interna, Gastroenterologia e Oncologia Medica, Dipartimento di Scienze Gastroenterologiche, Endocrino—Metaboliche e Nefro-Urologiche, Fondazione Policlinico Universitario A. Gemelli IRCCS, Università Cattolica del Sacro Cuore, 00168 Rome, Italy; giacintoabele.miggiano@unicatt.it; 2 UOC di Medicina Interna e Gastroenterologia, Area Medicina Interna, Gastroenterologia e Oncologia Medica, Dipartimento di Scienze Gastroenterologiche, Endocrino—Metaboliche e Nefro-Urologiche, Fondazione Policlinico Universitario A. Gemelli IRCCS, Università Cattolica del Sacro Cuore, 00168 Rome, Italy; franco.scaldaferri@policlinicogemelli.it (F.S.); marco.pizzoferrato@hotmail.it (M.P.); antonio.gasbarrini@unicatt.it (A.G.)

**Keywords:** vitamin D deficiency, Crohn’s disease, ulcerative colitis, BMI, phase angle

## Abstract

Hypovitaminosis D is frequently present in inflammatory bowel disease (IBD) with a higher incidence in Crohn’s disease (CD) than in Ulcerative Colitis (UC). Given the involvement of the alimentary tract, many factors can contribute to hypovitaminosis D. The aim of the study was to investigate the association of disease activity, body mass index (BMI) and phase angle with vitamin D deficiency in patients with IBD. A cross-sectional study was conducted on a cohort of 206 IBD patients (October 2016–September 2018). Of these patients, 32.6% were affected by hypovitaminosis D (CD: 38.6%; UC: 25.6%; *p* < 0.01). Negative and significant associations (*p* < 0.01) were found between BMI and vitamin D serum levels both in CD and UC patients. BMI represented a determinant of hypovitaminosis D (Odds Ratio (OR) = 1.12, *p* < 0.01) only in UC patients; phase angle was associated to hypovitaminosis D in both groups (CD: OR = 0.64, *p* < 0.05; UC: OR = 0.49, *p* < 0.01). Results of the present study confirm a higher incidence of hypovitaminosis D in patients with CD than in those with UC, and show that nutritional status plays a crucial role in the incidence of vitamin D deficiency in patients with IBD.

## 1. Introduction

Inflammatory bowel disease (IBD) is a chronic relapsing–remitting systemic disease of the gastrointestinal tract [1,2], characterized by an inflammatory process that requires lifelong treatment [3]. Crohn’s disease (CD) and Ulcerative Colitis (UC) are the two main types of inflammatory bowel diseases [4]. Crohn’s disease is a relapsing, transmural inflammatory disease of the gastrointestinal mucosa that can affect the entire alimentary tract, including the mouth, esophagus and stomach. Ulcerative Colitis is a relapsing non-transmural disease that affects colon and rectum [5,6,7].

The underlying causes of IBD are still unclear. However, extensive research demonstrates that both diseases are the result of a complex interaction among genetic, immunological and environmental factors [8,9], altered intestinal flora, a diet high in carbohydrates and fats, use of oral contraceptives, not breastfeeding, infections, vaccinations, antibiotics, pollution and industrialization are some of the most common risk factors in the onset of IBD [10,11].

Because of the increasing incidence of the disease in the healthy population [12], the disease chronicity and the involvement of the gastrointestinal tract, specific attention was dedicated to the evaluation of the nutritional status [3,13] of IBD patients to reduce the negative consequences derived by the prolonged disease activity [14], and to increase patient quality of life [15].

Health of the gastrointestinal tract is subject to several factors; multiple mechanisms can contribute to micronutrient deficiencies in IBD, such as decreased nutrient intake, increased intestinal loss, malabsorption, hypermetabolic state, drug interaction and long-term total parental nutrition [16]. In this context, vitamin D, a fat-soluble vitamin, plays a central role. In fact, vitamin D, beyond its classic role in calcium and bone homeostasis, plays a dynamic role in regulation of the immune system [17]. In the pathogenesis of immune-mediated diseases such as IBD, vitamin D is considered a paramount element [18,19]. Incidence of hypovitaminosis D is higher in patients with IBD (from 16% to 95%) than in the healthy population [20], and it is higher in patients with Crohn’s Disease than in those with Ulcerative Colitis [21].

In general, disease and non-disease related factors may lead to vitamin D deficiency. These may include malabsorption, impaired conversion of vitamin D into its active metabolites, increased metabolism of vitamin D, low exposure to sunlight, insufficient physical activity, reduced vitamin D in diet, and obesity [22,23]. Therefore, there is a bidirectional relationship between IBD and hypovitaminosis D [19]. On the one hand, low levels of vitamin D registered at the diagnosis suggest that vitamin D deficiency could increase the incidence of IBD in the healthy population [24], especially if we consider the crucial role of vitamin D as a regulator of the immune system [25]. On the other hand, vitamin D deficiency could be the combined effect of factors either unleashed by the inflammation itself or derived from the disease [26]; they are confounding factors which could persist in all stages of the disease, even in remission, and could be correlated with the disease itself (e.g., as in the case of malabsorption), its therapies, or patients’ lifestyle [15].

The concurrence of IBD and vitamin D deficiency is often observed at diagnosis and in the presence of a disease relapse. Many studies suggest that vitamin D deficiency can be both a determinant factor in the disease’s progression (and even one of the risk factors on the onset of the disease) [27,28,29] and an outcome of the disease itself [20,21]. Conflicting results have emerged about this association; while some studies reported a strong association between disease activity and vitamin D deficiency [3,30] even in remission [31], others demonstrated a lack of a statistically significant association [32,33]. These conflicting results could arise from the simultaneous study of Crohn’s disease and Ulcerative Colitis. In fact, while the association between Crohn’s disease and vitamin D deficiency is clear [26,30,34], in Ulcerative Colitis this association is not so clear. Some research demonstrated that disease activity and vitamin D levels are not associated in patients with UC [33,35], while other studies registered a significant association between vitamin D deficiency and disease activity in patients with UC [36]. In addition, low serum levels of vitamin D can increase the clinical relapse risk in patients with UC because of a drop in the immunoprotective and anti-inflammatory properties of vitamin D [31]. This indicates that it is necessary to further investigate the association between vitamin D deficiency and ulcerative colitis.

Furthermore, with specific attention to vitamin D, it is possible to identify many other factors affecting vitamin D serum levels; some of them are linked with the environment, such as the season or the latitude, while others correlate with some specific patient characteristics, like age, gender or anthropometric values [37,38]. In the specific case of IBD, analyzing vitamin D levels while taking environmental factors into consideration reveals in many studies that vitamin D levels are higher in summer than in winter, and that the incidence of hypovitaminosis D is higher in the Northern than in the Southern population [1,2,21,27,39,40].

Besides, the inflammation processes can impact on patients’ nutritional status, thus determining the severity of the underlying disease [41,42]. Since many studies have proven an association between body mass index and vitamin D levels [38,43,44], we propose that it is necessary to deepen the nutritional aspect when evaluating the impact of body mass index (BMI) on vitamin D levels and we also suggest introducing a bioimpedentiometry parameter such as the phase angle. While the association between BMI and vitamin D was studied in patients with IBD [17], the phase angle is never used in the gastroenteric field, nor in IBD patients. Phase angle is an indicator of fluid distribution in tissues and the electrical capacity of the human body; its measurement is performed through a bioelectrical impedance analysis (BIA) [42]. Cell mass and the integrity of cell membranes depends on patient characteristics such as age, BMI, or fluids distribution [43]. They are also influenced by the presence of inflammation, undernourishment status and prolonged physical inactivity, because these conditions negatively affect electrical tissue properties which determine a drop of phase angle values, especially when compared with those of healthy subjects [45]. Thus the phase angle, a biomarker of nutritional status, is a parameter useful for monitoring disease progressions [44]. Studies conducted in different medical fields (such as liver and renal disease, and oncology), in which phase angle was used as a measure of patients’ nutritional status, proved that there is a negative association between low values of phase angle and disease outcome [42,46,47,48].

This study had a threefold aim. The first was to investigate the association between disease activity and vitamin D deficiency in patients with IBD, comparing results between patients with UC and those with CD to determine the concrete difference among these groups, taking into account the genetic and immunologic differences in the pathogenesis of ulcerative colitis and Crohn’s disease. The second goal was to evaluate the impact of anthropometric and bioimpedentiometry factors such as BMI and phase angle on vitamin D levels, identifying any potential association between them. Finally, the third aim was to estimate the probability to develop vitamin D deficiency in conjunction with the nutritional status of the patient and with regard to the specific disease.

## 2. Materials and Methods

### 2.1. Patients

A cross-sectional study was performed on a cohort of 206 patients from October 2016 to September 2018 at the nutritional clinic department of the Fondazione Policlinico Universitario “A. Gemelli” IRCCS.

To be included in the study, patients were required to be affected by an inflammatory bowel disease and not hospitalized. For each patient, weight and height were measured to calculate BMI, while resistance and reactance were recorded to determine the phase angle value. Blood samples were collected concomitantly with anthropometric and bioimpedentiometry evaluations. None of the patients received vitamin D supplements. This project was part of a more complex study approved by the Ethics Committee of the Fondazione Policlinico Universitario “A.Gemelli” IRCCS (Prot. 28683/19—ID 2673).

### 2.2. Measurements

BMI was calculated as weight (kilograms) divided by the square of height (meters). Patients were classified for their BMI according to the World Health Organization (WHO) recommendations [47] as having a normal weight (18.5–24.9 kg/m^2^), being overweight (25.0–29.9 kg/m^2^) or obese (>30 kg/m^2^). Obesity categories were categorized as Class I (30–34.99 kg/m^2^), Class II (35–39.99 kg/m^2^), and Class III (>40 kg/m^2^) according to the report of the WHO consultation on prevention and management of obesity [47]. Phase angle was calculated as the arctangent of the ratio of reactance to resistance [46]; reactance and resistance values were assessed by bioelectrical impedance analysis (BIA) through a dedicated analyzer (BIA101 Anniversary, Akern, Italy).

Hemoglobin, C-Reactive Protein (CRP), albumin, ferritin, iron, folates and vitamin B12 levels were measured only for the purpose of comparing the two groups of patients (UC and CD) and to identify possible significant differences. The hemoglobin thresholds for men were as follows: <13 g/dL “low”, 13–17 g/dL “normal”, >17 g/dL “high”. For women: <12 g/dL “low”, 12–15 g/dL“normal”, >15 g/dL “high”. Iron ranges for men were as follows: <60 µg/dL “low”, 60–160 µg/dL “normal”, >160 µg/dL “high”. For women: <40 µg/dL “low”, 40–150 µg/dL “normal”, >100 µg/dL “high”. Vitamin B12 thresholds for both men and women were as follows: <187 pg/mL “low”, 187–883 pg/mL “normal”, >883 pg/mL “high”.

Disease activity and vitamin D serum levels were measured on the same day. To evaluate disease activity, we considered two indexes: The Partial Mayo Score and Harvey–Bradshaw index (HBI). The first was used to determine disease activity in patients with UC, and the second was used to evaluate the disease activity in patients with CD. Using the Partial Mayo Score and the Harvey–Bradshaw score, we divided patients into four groups: remission, mild, moderate and severe [49]. Cut-offs were reported in Table 1.

Vitamin D levels were determined through the measurement of serum levels of 25 hydroxyvitamin D (25(OH)D) by a chemiluminescence immunoassay (CLIA) [50]. According to Clinical Practice Guidelines from the US Endocrine Society, vitamin D deficiency is defined as serum 25-OH D less than 20 ng/mL (below 50 nmol/L) [51]. Values between 21–29 ng/mL indicate vitamin D insufficiency, while serum levels from 30 to 50 ng/mL are considered sufficient vitamin D serum levels. Values higher than 150 ng/mL indicate vitamin D poisoning [52,53]

### 2.3. Statistical Analysis

Data were recorded in an Excel File (Microsoft Corp., Redmond, WA, USA) and analyzed with STATA15 (Stata Corp., College Station, TX, USA). Descriptive statistics were calculated to identify the sample composition; qualitative variables were presented with frequencies and percentages, while quantitative variables were summarized using mean and standard deviation. For blood exam comparisons between UC and CD patients, chi-square tests were used for qualitative variables and the Mann–Whitney U test was performed for quantitative variables, given the non-normal distribution of data. Correlations between variables were verified using Spearman’s rho coefficient. Finally, a binary logistic regression was performed using as dependent variable vitamin D serum levels (1 if serum levels were lower than 20 ng/mL, and 0 otherwise); BMI, phase angle, disease activity and gender were used as independent variables. Both the Spearman correlation and logistic regression were performed for each group (UC and CD), separately. Statistical significance was set at 5% (*p* < 0.05).

## 3. Results

The sample included 206 patients showing IBD: 49.0% of them were affected by Crohn’s disease (CD), and 51.0% were affected by Ulcerative Colitis (UC). Of the patients, 49.5% were female and 50.5% were male; a female prevalence was registered in CD group (51.5%), while a male prevalence was observed in UC group (52.4%). The mean age at diagnosis was 36.7 ± 15.6 years (CD: 37.9 ± 16.6 years, UC: 35.7 ± 14.7 years), while mean age at the last follow-up was 44.1 ± 15.8 years (CD: 44.0 ± 16.3 years, UC: 44.2 ± 15.3 years). Non-smokers made up 52.9% of the sample, while 22.1% were smokers and 25.0% were former-smokers; there was no statistically significant differences between UC and CD patients concerning smokers and non-smokers. BMI was 24.03 ± 5.7 kg/m^2^ (CD: 24.4 ± 5.9 kg/m^2^; UC: 23.7 ± 5.4 kg/m^2^) without any significant difference between the two groups. the mean BMI, was not significantly different between CD and UC patients. Among those sampled, 64.8% had folates higher than 4 ng/mL with a significant difference (*p* = 0.017) between patients with UC (76.1%) and those with CD (59.0%). Patients with CD had a significantly higher prevalence of iron deficiency (48.5%) than those with UC (34.9%, *p* = 0.031). Vitamin B12 levels were significantly lower in CD patients (22.5%) than in UC patients (1.1%, *p* < 0.01). All descriptive statistics are reported in Table 2.

A significantly longer disease duration was observed in patients with UC (8.5 ± 8.4 years) compared to patients with CD (7.3 ± 5.5 years, *p* = 0.016). The whole sample was heterogenous concerning disease activity: 27.1% of patients were in remission, 34.0% displayed mild disease activity, 16.8% of patients presented moderate disease activity, and 22.8% of the sample displayed severe disease activity. (Table 3).

**Figure 1:**
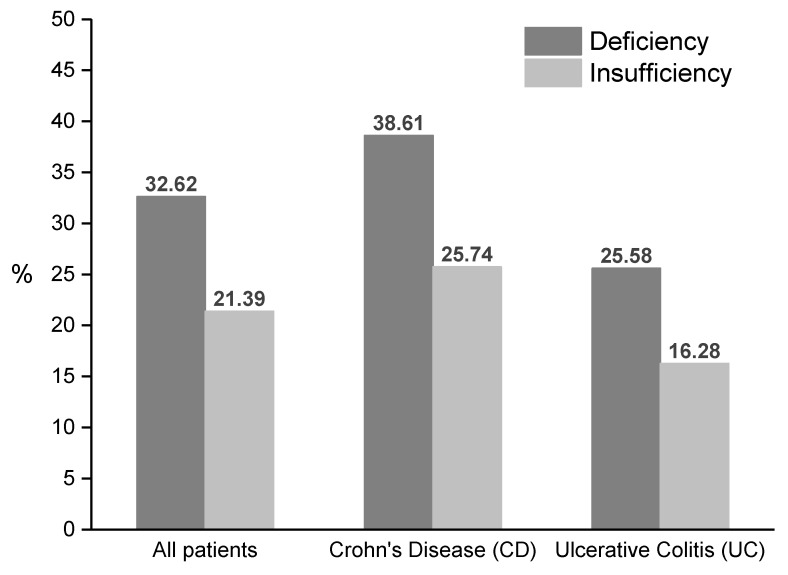
Percentages of vitamin D deficiency and insufficiency related to total sample (*n* = 206), Crohn’s disease (*n* = 101) and Ulcerative Colitis (*n* = 105).

Vitamin D deficiency and insufficiency were present in 32.6% and 21.4% of the sample, respectively, with a significantly (*p* < 0.01) higher incidence in patients with CD (deficiency: 38.6%; insufficiency: 25.7%) in comparison with UC patients (deficiency: 25.6%; insufficiency: 16.3%); (Figure 1).

A statistically significant association (*p* = 0.001) was observed between disease activity and vitamin D deficiency in patients with CD (Table 4); particularly in the presence of remission, vitamin D deficiency was registered in 42.9% of CD patients. Hypovitaminosis D was observed in 20% of the CD patients with mild disease activity. High percentages of vitamin D deficiency were registered in CD patients with moderate (78.6%) and severe disease activity (47.1%). No statistically significant association between disease activity and vitamin D deficiency was observed in patients with UC, although patients presenting moderate or severe disease activity displayed a higher prevalence of vitamin D deficiency than those experiencing mild disease activity. A significant association between BMI classes and vitamin D deficiency was observed for UC patients (Table 4, *p* = 0.013): hypovitaminosis D was found in 62.5% of obese patients in class I and for 100% of obese patients in class III. Despite being statistically significant, this difference must be considered with great care, as the number of patients included in the obesity classes are not enough to draw strong conclusions.

**Figure 2:**
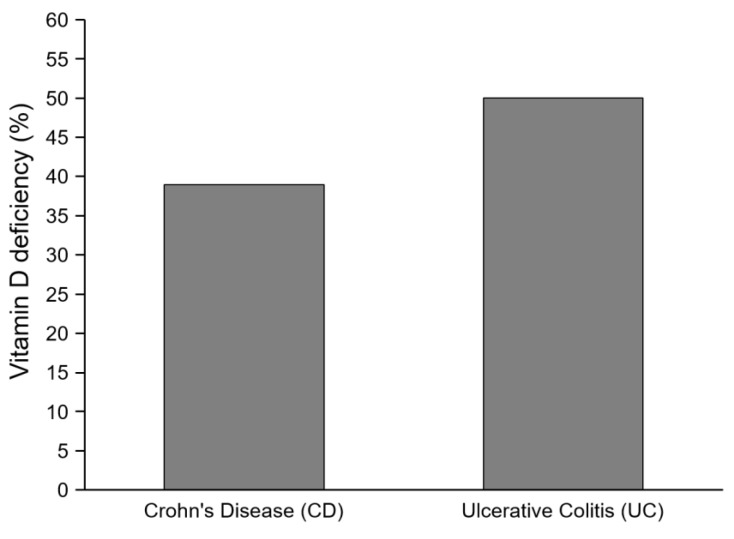
Percentages of patients with vitamin D deficiency.

When analyzing patients who were diagnosed with the disease less than 12 months before the measurement, no significant differences in vitamin D deficiency were found between UC and CD patients: 38.9% of patients with CD were affected by hypovitaminosis D compared to 50% of the UC patients (*p* > 0.05, Figure 2). When considering vitamin D deficiency and insufficiency jointly, it emerged that 50% and 75% of patients with CD and UC, respectively, presented at least a vitamin D insufficiency (*p* > 0.05).

No statistically significant differences in hypovitaminosis D were observed (*p* > 0.05) between patients with a recent diagnosis (less than 12 months) and those with a disease duration longer than 12 months: 42.3% vs 31.1%.

**Figure 3:**
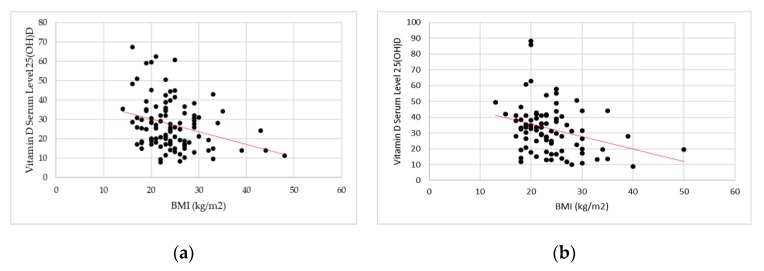
Scatterplot—correlation between body mass index (BMI) and vitamin D serum levels 25(OH)D: (**a**) Crohn’s disease patients; (**b**) Ulcerative Colitis patients.

Significant results emerged in the study of correlation between BMI and vitamin D serum levels in both groups. Spearman’s coefficient showed a negative and weak correlation between BMI values and vitamin D serum levels (CD: *r*_s_ = −0.2257, *p* = 0.0038; UC: *r*_s_ = −0.2847, *p* = 0.0079) (Figure 3).

A negative correlation between disease duration and vitamin D serum levels was observed in patients affected by Crohn’s Disease (*r*_s_ = −0.1381, *p* = 0.1685), although it was not statistically significant. A positive correlation between disease duration and vitamin D serum levels in patients with UC (*r*_s_ = 0.0646, *p* = 0.5544) was found, although also in this case it was not statistically significant. No statistically significant correlations were registered between the phase angle and vitamin D serum levels in both groups (CD: *r*_s_ = 0.1738, *p* = 0.0837; UC: *r*_s_ = 0.0699, *p* = 0.5249).

The probability of developing a vitamin D deficiency was separately analyzed in both groups of patients through a logistic regression (Table 5). Specifically, in patients with CD, a decrease of phase angle value implied a rise of 40% in the likelihood of developing a vitamin D deficiency (Odds Ratio, OR = 0.66, *p* < 0.05). The progression from a mild disease activity to a moderate disease activity was associated with a rise of 92% in the likelihood of developing a vitamin D deficiency (OR = 11.22, *p* < 0.01) in patients affected by CD. For CD patients in remission, the risk of developing a vitamin D deficiency increased to 77% if it was compared with the risk of developing a hypovitaminosis D with mild disease activity. In patients with UC, the binary regression demonstrated that BMI value had a positive impact on vitamin D deficiency (OR = 1.12, *p* < 0.01); an increase of 1 point in BMI was associated with a rise of 12% in the likelihood of developing vitamin D deficiency. In patients with UC, the phase angle had a negative impact on the probability of developing a hypovitaminosis D (OR = 0.49, *p* < 0.01), demonstrating that an increase in the phase angle value was associated with a 33% decrease in the likelihood to develop vitamin D deficiency. The disease progression was not a risk factor in patients with UC. All logistic regression models were validated by a likelihood-ratio(LR) chi-squared test, for which the *p-*value remained lower than 0.01.

## 4. Discussion

The aim of this cross-sectional study was to compare vitamin D deficiency in a cohort of 206 patients affected by IBD, distinguishing between patients with UC and with CD respectively, to evaluate the association between vitamin D deficiency and some disease and non-disease parameters such as disease activity, BMI and phase angle, and to determine the risk of these factors on the development of vitamin D deficiency. Another aim of the study was to determine vitamin D deficiency over the 12 months from diagnosis in comparison with vitamin D deficiency reported by patients with a longer disease duration, to verify whether low vitamin D levels could be considered a concomitant cause in the onset of IBD.

Crohn’s disease and Ulcerative Colitis—chronic intestinal diseases characterized by inflammation of the bowel—are the primary constituents of inflammatory bowel disease. The causes of IBD are still unclear; on the one hand (direct relationship), the onset of IBD seems due to an interaction between genetic and environmental risk factors, vitamin D deficiency included [19], and on the other hand (inverse relationship), low vitamin D levels could be due to an interaction between some specific disease-related variables, or to a variation in dietary habits or in lifestyle, changes linked with the disease itself in any case [11].

In this study we registered a vitamin D deficiency of 54%, value similar to that found by Ulitski et al. and Burrelli Scotti et al. for who hypovitaminosis D incidence was of 49.8% and 59.8% respectively [15,54]. Nevertheless, the observed percentage value was particularly high if we consider that this study was conducted on a cohort of Italian patients for whom geographical latitude is not considered a risk factor in the onset of vitamin D deficiency, as in other countries [40,55,56], and when comparing our results with those achieved by Burrelli Scotti in their control group (30% of incidence) and Lippi in the general population (36% of incidence) [52,55]. However, the incidence of vitamin D deficiency observed in our study was lower than that reported by a recent Ko’s study, in which they demonstrated as hypovitaminosis D is higher in patients with IBD (73.6%) than in the general Korean population [33].

The onset of IBD typically occurs in the second and third decades of life [56], although some recent studies demonstrated an increase of IBD incidence among young people due to an increase of risk factors such as pollution, lower exposure to sunlight and a diet highs in fats (i.e., “bad” dietary habits like the consumption of “junk food”) [56,57].

### 4.1. Direct Relationship: Vitamin D Deficiency as Risk Factor in the Onset of IBD or in the IBD Progression

Many epidemiologic studies suggest that vitamin D deficiency plays a crucial role in the onset of IBD and in the progression of IBD itself [9,58]. In particular, low serum levels of vitamin D could be among the concomitant causes in the onset of IBD, as demonstrated by many European studies [40,54,55], from which it emerged that in Northern regions where the sunlight exposure and the natural synthesis of vitamin D are lower, the incidence of CD and UC are relatively higher than the 80% and 40% found in the Southern regions [52]. Vitamin D deficiency plays a paramount role in the disease progression too [3,15,36], because vitamin D levels impact functions of the gut barrier and immune system [1]. The prevalence of hypovitaminosis D in the disease progression was confirmed by Sadeghian’s study (2016), which demonstrated that there is greater disease activity in concomitance of a decrease of 25 (OH)D concentrations [57]. In our study, no statistical significance of vitamin D deficiency was observed within the first 12 months from the diagnosis, thus showing that there is no evidence of vitamin D deficiency in the onset or progression of IBD in the first 12 months of disease, as just proved by Hassan [59].

### 4.2. Inverse Relationship: Onset Of Hypovitaminosis D In Patients with IBD

Hypovitaminosis D can be determined by several factors, related or unrelated to the disease. The association between IBD and vitamin D deficiency is related to environmental factors such as pollution, the latitude of patients’ residence or season, or to factors linked with patients’ lifestyle, such as reduced physical activity and outdoor activities, leading to a lower exposure to sunlight. Some changes of life habits are correlated with IBD symptomatology, pharmacological treatments or side-effects related to medicine assumption. In the long term, vitamin D deficiency can be the consequence of malabsorption in the small intestine and the direct effect of the intestinal inflammation or chirurgical resection. We showed that vitamin D deficiency is higher in patients with CD than in those with UC (64% in CD and 54% in UC), thus confirming the results reported by several studies [35,58], which demonstrated that serum levels of 25 (OH)D were lower in patients with CD than in those with UC, and that this difference was due to a malabsorption of vitamin D in the small intestine because of inflammation activity [21,28].

Concerning disease activity, it emerged that patients with Crohn’s disease in remission presented a higher vitamin D deficiency (43% of incidence) than patients with UC (23% of incidence). In both groups, hypovitaminosis D was higher in remission than in mild disease activity. This finding could be explained by several concomitant factors, including the complex clinical history of these patients. Nevertheless, additional studies are warranted to further explore this matter. Even though previous studies demonstrated that serum levels of 25 (OH)D were higher in CD patients in remission than in CD patients with a mild or moderate disease activity [27,34,59,60,61,62], they did not distinguish between mild and moderate disease activity. In fact, it was demonstrated that the capacity of vitamin D absorption is reduced of 30% on average in CD patients in remission with respect to healthy subjects, even if a supplementation of vitamin D is administered, so that vitamin D deficiency or insufficiency is greater of 70% in patients with CD in remission [60] because of a malabsorption problem [19].

As to disease activity, we confirmed many of the results achieved in previous studies. In the presence of active disease, vitamin D deficiency is prevalent in patients with CD, especially in those patients with a moderate disease activity (79% of CD patients). Results showed a significant difference among the four stages of disease, as demonstrated by Jorgensen, O’Sullivan and Garg’s studies [8,23,34]. In patients with UC, vitamin D deficiency is higher in patients with a moderate disease activity (41% of incidence); a greater incidence of hypovitaminosis D in these patients may not be correlated with malabsorption problems as seen for Crohn’s disease, but it can be the result of the drugs used in the treatment of UC [36] and dietary and behavioral restrictions. However, it is necessary to note that the effect of disease activity on vitamin D deficiency in patients with UC is smaller (no statistical significance) in comparison with that observed in CD patients, for which the malabsorption significantly affects disease activity.

In this context, we observed that disease activity represents a potentially determinant factor in patient with CD so that a moderate disease activity could increase the likelihood to develop hypovitaminosis D of 92%. As demonstrated by Burrelli Scotti, we observed that patients with CD are subjected to a greater risk of hypovitaminosis D than patients with UC, although in our study we noted that remission itself represents a risk factor in the develop of vitamin D deficiency, with an impact of 77% in the development of hypovitaminosis D in comparison with patients in a mild disease phase [54].

The data here presented suggest that hypovitaminosis D could be associated with disease activity, which is consistent with several other studies [15,21,27].

The analysis of a possible association between anthropometric values and vitamin D deficiency showed a uniform incidence of hypovitaminosis D in patients with CD without any difference among different BMI classes (32% in patients with a normal weight, 31% in underweight patients, 50% in obese class I, 50% in obese class II, and 67% in obese class III), although an increase in BMI values, and therefore a tendency to obesity, could produce a decrease in vitamin D serum levels in patients with CD. The negative and weak correlation observed between these two variables underlines that there could be situations in which a BMI increase and a reduction of vitamin D levels do not necessarily cause an immediate vitamin D deficiency; nevertheless, this indicates that in the long run an unmonitored increase in BMI values could determine hypovitaminosis D even in patients not initially affected by this deficiency. It could be argued that vitamin D deficiency, given its association with the disease activity, may also correspond to a lower BMI index. Indeed, diarrhea, gastrointestinal bleeding and abdominal pain in IBD patients often lead to weight loss. However, the inverse relationship between BMI and vitamin D that we found may suggest that a higher BMI does not necessarily correspond to a normal nutritional condition.

In patients with UC, obesity seems to be associated with vitamin D deficiency (obesity class I: 63% of incidence, obesity class II: 33% of incidence, and obesity class III: 100% of incidence); low percentages of incidence were identified in underweight patients (25%) and in those with a normal weight (14%). For UC patients, an increase of BMI values produces a rise of 12% in the likelihood to develop hypovitaminosis D as demonstrated by Pallav and ESPEN guidelines [44,62].

However, since the number of patients included in the different obesity classes was limited, our results on the potential association between obesity and vitamin D deficiency do not allow to draw definitive conclusions. Future studies on a larger number of obese patients should be carried out to confirm our preliminary results.

In both groups of patients, we have shown that the phase angle—a bioimpedentiometry parameter of the cell membrane integrity and distribution of water among intra- and extra-cellular spaces—may predict the probability of developing vitamin D deficiency. Indeed, a one-point decrease in the phase angle increases the likelihood of hypovitaminosis D of 40% in patients with CD and 33% in patients with UC, respectively. This is consistent with another study showing that phase angle has the potential to be a biomarker of inflammation and a tool to possibly identify obese IBD patients who could benefit from vitamin D supplementation [63].

In accordance with the Vimaleswarns and Pereira-Santos’ studies, which demonstrated a direct relationship between obesity and vitamin D deficiency independent of other factors such as age or latitude, the findings reported in our study, suggest that in IBD patients, vitamin D deficiency could be associated with intestinal inflammation and the related therapeutic treatments, which may lead to malabsorption or reduced synthesis of vitamin D [38,64]. Besides, anthropometric and bioimpedentiometry characteristics could affect vitamin D deficiency along with disease activity. We conclude that vitamin D deficiency, or the risk of developing it, could be present in patients with IBD, even in those cases in which known environmental factors are absent (e.g., low exposure to sunlight) [23].

To our knowledge, this is the first study in which a bioimpedentiometry parameter, such as the phase angle, is used to determine the risk of hypovitaminosis D in patients with IBD.

Our study presents some limitations. For each pathology, we used only one score to identify disease activity, without performing a thorough evaluation on the outcome of each patient. Indexes being used were mainly clinical, such as the Partial Mayo Index for UC and the Harvey Bradshaw Index for CD; biomarkers of inflammation being measured were limited to CRP levels. Further studies should therefore aim at a better biochemical characterization of the patient’s inflammatory status. Similarly, further studies should address questions concerning how potential confounders, such as IBD medications, IBD-related surgeries, smoking status and family history may affect the association between vitamin D and IBD disease activity. The use of medications should be assessed especially for their potential role in inducing hypovitaminosis D. Concerning pure clinical observations, future studies on remitting patients should include data on the more advanced stages of the disease before remission; further, they should describe and investigate alternative IBD outcomes (such as hospitalizations, required surgeries, medication failure rates). Additionally, a prospective study should be carried out to evaluate the incidence of hypovitaminosis D in the onset of IBD or its progression. Moreover, we did not consider the potential effects of seasonality on vitamin D levels. In our opinion, however, the potential impact of seasonality on the results is limited, as the patients were assessed homogeneously within the study period. Finally, the present study is observational; therefore, it cannot establish causation between IBD disease activity, BMI, vitamin D deficiency and phase angle. This should be the subject of future investigations with different study design.

## 5. Conclusions

The reported associations between vitamin D deficiency and the parameters analyzed highlight a potential interaction between IBD and vitamin D deficiency. IBD seems to have a direct impact on vitamin D deficiency as a consequence of malabsorption, especially in patients with CD. Risk of hypovitaminosis D is high also in patients with UC, for which some pharmacological therapies may cause malabsorption. A sedentary lifestyle and poor dietary habits can have an impact on BMI and body composition in IBD patients, thus influencing vitamin D serum levels. The association between IBD and vitamin D deficiency indicates that IBD patients may benefit from a continuous monitoring and, when needed, vitamin D supplementation, especially in those patients exhibiting anthropometric (BMI) or bioimpedentiometry (phase angle) parameters able to predict vitamin D levels.

## Figures and Tables

**Table 1:** Mayo Score and Harvey–Bradshaw index classification.

Disease Activity	UC (Partial Mayo Score)	CD (Harvey-Bradshaw Index)
Remission	<2	<5
Mild Disease	2–4	5–7
Moderate Disease	5–7	8–16
Severe Disease	>7	>16

UC: Ulcerative Colitis; CD: Crohn’s disease.

**Table 2:** characteristics. Results displayed as the number of patients (percentage of the total number of patients for which a given parameters was measured). Body mass index (BMI) and phase angle are displayed as mean (standard deviation). Some of the parameters listed in the table were measured in a subset of the total number of patients.

Parameter	Total (*n* = 206)	CD (*n* = 101)	UC (*n* = 105)	*p*
**Gender (%)**				
Female	102 (49.51)	52 (51.49)	50 (47.62)	0.579
Male	104 (50.49)	49 (48.51)	55 (52.38)	
**Age**				
At diagnosis, mean (SD	36.78 (15.66)	37.9 (16.64)	35.7 (14.65)	0.4606
At the last follow-up, mean (SD)	44.1 (15.77)	43.96 (16.33)	44.24 (15.28)	0.8498
**Smoke (%)**				
Non Smoker	36 (52.94)	13 (50.00)	23 (54.76)	0.354
Smoker	15 (22.06)	8 (30.77)	7 (16.67)	
Former Smoker	17 (25.00)	5 (19.23)	12 (28.57)	
**Body Mass Index (BMI, kg/m^2^), mean (SD)**	24.03 (5.68)	24.43 (5.92)	23.66 (5.44)	0.3192
**Phase Angle, mean (** **SD** **)**	5.41 (1.00)	5.40 (1.00)	5.50 (1.00)	0.615
**Glycemia (%)**				
<65 mg/dL	57 (30.65)	27 (29.03)	30 (32.26)	0.868
65–110 mg/dL	118 (63.44)	60 (64.52)	58 (62.37)	
>110 mg/dL	11 (5.91)	6 (6.45)	5 (5.38)	
**Hemoglobin (%)**				
low	23 (12.71)	12 (13.04)	11 (12.36)	0.608
normal	154 (85.08)	77 (83.70)	77 (86.52)	
high	4 (2.21)	3 (3.26)	1 (1.12)	
**CRP (%)**				
<5.0 mg/L	133 (72.28)	67 (72.04)	66 (72.53)	0.941
>5.0 mg/L	51 (27.73)	26 (27.96)	25 (27.47)	
**Total Proteins (%)**				
<65 g/L	5 (2.87)	3 (3.53)	2 (2.25)	0.573
65–85 g/L	153 (87.93)	76 (89.41)	77 (86.52)	
>85 g/L	16 (9.20)	6 (7.06)	10 (11.24)	
**Albumin (%)**				
<34 g/L	10 (6.02)	7 (8.54)	3 (3.57)	0.254
34–48 g/L	155 (93.37)	75 (91.46)	80 (95.24)	
>48 g/L	1 (0.60)	-	1 (1.19)	
**Folates (%)**				
<4 ng/mL	55 (32.16)	34 (40.96)	21 (23.86)	0.017
>4 ng/mL	116 (67.84)	49 (59.04)	67 (76.14)	
**Iron (%)**				
low	72 (43.11)	49 (48.51)	23 (34.85)	0.031
normal	92 (55.09)	52 (51.49)	40 (60.61)	
high	3 (1.80)	-	3 (4.55)	
**Vitamin B12 (%)**				
low	23 (12.11)	22 (22.45)	1 (1.09)	0.000
normal	158 (83.16)	72 (73.47)	86 (93.48)	
high	9 (4.74)	4 (4.08)	5 (5.43)	

SD: standard deviation; CRP: C-reactive protein.

**Table 3:** Characteristics.

Parameter	Total (*n* = 206)	CD (*n* = 101)	UC (*n* = 105)	*p*
**Disease duration (years), mean (SD)**	7.33 (7.96)	6.06 (7.33)	8.54 (8.39)	0.016
**Disease Activity (%)**				
Remission	55 (27.09)	28 (28.28)	27 (25.96)	0.145
Mild	69 (33.99)	40 (40.40)	29 (27.88)	
Moderate	34 (16.75)	14 (14.14)	20 (19.23)	
Severe	45 (22.17)	17 (17.17)	28 (26.92)	
**Body-mass Index (BMI) (%)**				
underweight	26 (12.62)	13 (12.87)	13 (12.38)	0.964
normal weight	101 (49.03)	47 (46.53)	54 (51.43)	
pre-obesity	53 (25.73)	28 (27.72)	25 (23.81)	
obesity class I	16 (7.77)	8 (7.92)	8 (7.62)	
obesity class II	5 (2.43)	2 (1.98)	3 (2.86)	
obesity class III	5 (2.43)	3 (2.97)	2 (1.90)	

**Table 4:** Association between disease activity, BMI and Vitamin D deficiency.

Parameter	Chron’s Disease (*n* = 101)			Ulcerative Colitis (*n* = 105)		
	Vitamin D Deficiency	No Vitamin D Deficiency	*p*	Vitamin D Deficiency	No Vitamin D Deficiency	*p*
**Disease Activity (%)**						
Remission	42.9	57.1	0.001	22.7	77.3	0.106
Mild	20.0	80.0		9.1	90.9	
Moderate	78.6	21.4		41.2	58.8	
Severe	47.1	52.9		33.3	66.7	
**BMI classes (%)**						
underweight	30.8	69.2	0.625	25.0	75.0	0.013
normal weight	31.9	68.1		14.3	85.7	
pre-obesity	46.4	53.6		26.3	73.7	
obesity class I	50.0	50.0		62.5	37.5	
obesity class II	50.0	50.0		33.3	66.7	
obesity class III	66.7	33.3		100.0	0.0	

**Table 5:** Regression.

Parameter	Crohn’s Disease—OR (SE)	Ulcerative Colitis—OR (SE)
**BMI (kg/m^2^)**	1.05 (0.04)	1.12 *** (0.05)
**Phase Angle**	0.66 ** (0.11)	0.49 *** (0.10)
**Disease Duration**	1.03 (0.04)	0.97 (0.04)
**Disease Activity**		
Remission	3.35 ** (2.04)	1.44 (1.10)
Moderate	11.22 *** (9.09)	2.67 (1.98)
Severe	2.23 (1.58)	2.69 (1.92)
**Gender (male)**	0.41 (0.22)	0.64 (0.38)
**LR chi2(5)**	25.89	28.81
***p*** **-value**	0.000	0.000
**Num. Obs**	98	84

OR: Odds ratio. SE: standard error. LR: likelihood ratio. Significance levels: ** *p* < 0.05, *** *p* < 0.01.
